# Agreement of Peru-Based Allergy Tests for Respiratory Allergens in Allergic Rhinitis Patients

**DOI:** 10.1155/2024/8657483

**Published:** 2024-08-20

**Authors:** César Galván, Margarita Totesaut, Edgar Muñoz, Rafael Durán, Christian La Rosa, Oscar Calderón

**Affiliations:** ^1^ Emedic Salud, Lima, Peru; ^2^ Pediatric Health National Institute, Lima, Peru; ^3^ Muñoz Laboratory, Arequipa, Peru; ^4^ Department of Clinical Pathology Dos de Mayo National Hospital, Lima, Peru; ^5^ Facultad de Medicina Universidad Nacional Mayor de San Marcos, Lima, Peru; ^6^ SANNA el Golf Clinic, Lima, Peru

## Abstract

**Background:**

The Peruvian Immunoblot panel, together with traditional skin prick tests (SPT), are widely used in vitro allergy tests in Peru. In addition to this, Peruvian allergists are increasingly adopting multiplex tests such as the ALEX-2 (Macro Array Diagnostics). Previous studies have revealed limited agreement between Immunoblot and SPT results. Therefore, our study aimed to evaluate the concordance between these three tests in patients with allergic rhinitis (AR) in a private center in Arequipa, Peru.

**Materials and Methods:**

We enrolled 35 patients, including children over 3 years and adults, with AR. Clinical and demographic data were collected, and patients underwent allergic sensitization testing using the Immunoblot Peruvian panel (32 allergens), ALEX-2 (295 allergens), and SPT (12 allergens). Concordance was calculated using Cohen's kappa coefficient and analyzed with IBM SPSS V26.

**Results:**

Among the patients, 34.3% exhibited moderate-to-severe persistent AR, and 14.3% had asthma. Additionally, 85.7% reported a family history of AR. Sensitization rates varied notably between the SPT and ALEX-2, particularly for olive pollen (34.3% vs. 17.4%), Blomia tropicalis (11.4% vs. 17.1%), and grasses (11.4% vs. 28.5%). Remarkably, these allergens were not included in the Peruvian Immunoblot panel. Concordance analysis included seven allergens and showed significant concordance between ALEX-2 and SPT for five allergens, between Immunoblot and SPT for two allergens, and between ALEX-2 and Immunoblot for two allergens.

**Conclusion:**

This preliminary study shows us a better concordance between ALEX-2 and SPT rather than between Immunoblot and SPT.

## 1. Introduction

Allergic rhinitis (AR), the most common form of respiratory allergy, has a global prevalence of up to 18.1%, with nonspecific rhinitis reaching 29.4% [1]. In Peru, the estimated prevalence of AR is very similar to the global prevalence, reaching up to 18% [2]. While more prevalent in high-income countries, AR is rapidly increasing in middle and low-income countries [3]. This rise has implications for both public health and economic burden due to its high prevalence, resulting in decreased productivity, reduced school attendance, and impaired sleep quality [4].

Due to this global growth trend, as well as potential economic loss, accurate diagnosis has become a crucial tool for effective disease management. Diagnosing AR can be achieved through a comprehensive review of the patient's medical history and physical examination. However, to attain a more precise diagnosis, allergen-specific IgE testing via skin prick test (SPT) or blood tests is often necessary and vital, especially when considering specific immunotherapy [5].

Of the available diagnostic tests, the SPT remains highly effective in identifying patients with potential sensitization to AR or allergic asthma [6, 7]. Furthermore, advancements in molecular allergy (MA), such as multiplex and singleplex assays, are revolutionizing diagnosis and treatment. These tools provide greater efficiency by simultaneously testing for numerous allergens [8]. One such test, the Allergy Explorer version 2 (ALEX-2) [9], along with antibody-specific tests like Immunoblot [10, 11], plays a crucial role in pinpointing allergen sensitivity. This precise identification guides treatment decisions, particularly the selection of allergen-specific immunotherapy.

MA tests can be conducted through the identification of a specific allergen, known as singleplex assays, or through multiple allergens, known as multiplex assays. Singleplex assays offer the opportunity to measure extracts and molecules in a single session and are usually more cost-effective when a solid diagnosis is already established. However, they may overlook potential sensitivities. On the other hand, multiplex assays provide information on a broader range of potential allergens or the patient's sensitization profile. Additionally, they are particularly useful when a wide array of possible allergic sensitivities needs to be identified [12].

SPT stands out as a reliable, cost-effective, and well-tolerated method for determining IgE sensitivity, involving a simple skin puncture procedure [13]. Immunoblot is used to detect specific antigens recognized by two types of antibodies: those naturally produced in the body, called polyclonal antibodies, and those generated in the laboratory, known as monoclonal antibodies [14]. ALEX-2 goes beyond simply identifying total IgE reactivity; it also analyzes the specific IgE response to allergen components and allergenic extracts [15].

Having a diverse array of available tests, this study seeks to assess the specific concordance of the SPT with the multiplex Immunoblot and ALEX-2 tests in patients suffering from AR. These participants were treated at a private medical center in Arequipa, Peru, during the year 2023.

## 2. Materials and Methods

### 2.1. Study Design and Patients

A cross-sectional observational study of patients diagnosed with AR was conducted to assess the concordance between three allergen identification tests. Data collection, including demographic variables and allergen testing, took place in the second half of 2023 in a private medical center in Arequipa, Peru. The allergens investigated were classified into respiratory and food allergens. Of these, food allergens were analyzed only for positivity and respiratory allergens for concordance. Within the category of respiratory allergens, we classified them into dust mites, molds, and allergens produced by animals or insects. The study did not influence the proper management of clinical practice, as it adhered to the established protocols of the medical center.

The sample size was calculated using Epidat Version 4.2 software, with a 95% confidence level, a 5% precision, and an expected Cohen's kappa value of 0.084, resulting in a total of 30 patients. The final sample consisted of 35 patients, selected by a systematic random sampling method, which included children over 3 years old up to adults with a median age of 9 years and an interquartile range of 6–30 years.

Regarding the inclusion criteria, patients suspected of having AR based on their medical history and physical examination were selected. Patients diagnosed with other respiratory diseases were excluded from the study. These patients were receiving treatment at the medical center for suspected allergies.

The present research was approved by the Ethics Committee of San Bartolomé Hospital in Lima, Peru. Upon arrival at the healthcare center, patients or their direct family members, in the case of minors, were informed about the research study and were offered the opportunity to participate in the study and undergo the three diagnostic tests at no cost on the same day, with a guarantee of data confidentiality. Informed consent was obtained from all participants or their parents/guardians.

### 2.2. Data Collection Techniques

Various techniques were employed to gather the necessary data for the study. Initially, a demographic questionnaire and epidemiological questions, such as family history, were conducted during medical interviews. Subsequently, SPT, Immunoblot, and ALEX-2 allergen tests were performed, and the results were recorded in a structured form. The SPT utilized 12 different extracts of respiratory allergens, which are the most frequently encountered in the Arequipa region, according to experts. The Immunoblot employed the Peruvian panel, consisting of 32 allergens, while 295 allergen extracts are used for ALEX-2.

The Peruvian Immunoblot panel does not contain the important allergens olive pollen, Blomia tropicalis, and grasses, and therefore, their sensitivities could not be evaluated with this test. For this reason, it was decided to perform the concordance analysis with seven allergens that all three tests have in common.

For the SPT, an allergenic solution was applied to the forearm skin, followed by a small puncture with a lancet. Blood samples were extracted from patients for the Immunoblot and ALEX-2 tests, which were processed in the medical center's laboratory. In the case of the Immunoblot, the blood sample was placed on a reactive strip with allergens, while for the ALEX-2 test, it was placed on a microarray with microspheres containing various allergens.

Results from the SPT were documented in a technical form as reactions occurred. These results, along with the laboratory results from the Immunoblot and ALEX-2 tests, were then digitized into a Microsoft Excel database systematically. Access to this database was restricted to researchers to maintain result confidentiality. It is important to note that allergen sensitization diagnoses were provided to individual patients and, in the case of minors, to a close relative.

### 2.3. Statistical Analysis

Statistical analysis was conducted using IBM SPSS version 26 software. Descriptive statistics were conducted to represent sociodemographic variables, along with determining the percentage of each allergen detected based on the respective allergen test utilized. Concordance between SPT, Immunoblot, and ALEX-2 was calculated for qualitative results, focusing on indications of positivity or negativity by the seven allergens detectable in common by the three tests. Cohen's kappa coefficient, along with positive and negative agreement, were assessed for each extract and molecule considered. Additionally, each method's qualitative agreement with the results of the SPT was evaluated. Cohen's kappa coefficient cutoff points were applied as follows: values between 0.01 and 0.20 indicated slight agreement; 0.21–0.40 suggested fair agreement; 0.41–0.60 indicated moderate agreement; 0.61–0.80 suggested substantial agreement, and 0.81–1.00 indicated nearly perfect or perfect agreement.

## 3. Results

### 3.1. Sociodemographic and Clinical Characteristics of the Patients

The sociodemographic and clinical characteristics of patients with AR were examined at a private center in Arequipa during 2023, as illustrated in Table 1. The study encompassed a total of 35 patients, with 21 identified as male subjects, constituting 60% of the sample. The age distribution shows that the median was 9 years, with a variability between 6 and 30 years.

Regarding the severity of AR allergies, the majority presented with either mild intermittent (28.57%) or moderate–severe persistent conditions (34.29%). Clinical results revealed a prevalence of conjunctivitis in 37.14% and 14.29% of patients with asthma and atopic dermatitis, respectively. Notably, family medical histories indicated a significant prevalence of AR (85.71%) among relatives.

In terms of treatment, more than half of the patients (51.43%) were prescribed antihistamines, while a smaller proportion received intranasal corticosteroids (17.14%). Despite the observed severity of allergies, only 37.14% of the patients demonstrated adherence to the prescribed treatments. No cases of food allergies were reported, while drug allergies were observed in a small proportion (2.86%) of the patients.

### 3.2. Allergen Detection

In Table 2, we present the positive results obtained with SPT, Immunoblot, and ALEX-2 for the detection of respiratory and food allergens. In particular, the highest percentage of positive detections in all three methods was observed for animal epithelial allergens. Canine epithelium, for example, showed significant positivity rates: 34.29% by SPT, 5.71% by Immunoblot, and a remarkable 62.86% by ALEX-2. Similarly, cat epithelium showed substantial positive detections, with rates of 25.71% by SPT, 11.43% by Immunoblot, and 51.43% by ALEX-2.

The results in this table also show higher detection rates with ALEX-2 for all allergen types with some exceptions like the case of *D. farinae*, which was detected mainly by SPT (22.86%) and Immunoblot (20.00%), and the pollen allergen Olive, were 34.3% of patients exhibited sensitization on the SPT, followed by 17.14% for ALEX-2. In addition, some allergens had specific sensitizations for a diagnostic test, such as the case of Salsola, which was present in 20.00% of patients using the ALEX-2 test.

Certain allergens exhibited low rates across all three methods. For instance, in the case of Cladosporium allergen, slightly higher percentages were observed for the SPT at 5.71%, compared to 2.86% for the other methods. In contrast, all three allergy tests produced consistent results with no variation for Blatella, despite showing sensitization in only one patient.

### 3.3. Concordance between Diagnostic Tests

Cohen's kappa coefficient revealed varying levels of agreement between ALEX-2 and SPT allergen identification tests (Table 3). Agreement ranged from fair (kappa = 0.381) for the mite allergens *D. pteronyssinus* to almost perfect (kappa = 0.822) for *D. farinae*. These findings suggest a fair and nearly perfect agreement, respectively, when compared to other assessed allergens.

The insect allergen Blatella concordance was the one that stood out with very close to 100% agreement between the two diagnostic tests (kappa = 0.999). Furthermore, cat epithelium revealed a moderate level of agreement (kappa = 0.493). Overall analysis using ALEX-2 and SPT revealed a statistically significant final agreement obtained for five of the seven allergens evaluated, with the exception of Cladosporium and dog epithelium.

In Table 4, the concordance analysis of ALEX-2 is presented, contrasted with Immunoblot. Varying levels of agreement were observed among allergens. For dust allergens, only *D. farinae* showed statistically significant substantial agreement (kappa = 0.717). Among mold allergens, Alternaria stood out with moderate concordance (kappa = 0.545).

Regarding animal allergens, dog epithelium and cat epithelium exhibited varied but statistically insignificant levels of concordance. The comprehensive analysis of concordance between ALEX-2 and Immunoblot yielded statistically significant results for only two allergens: *D. farinae* (dust allergen) and Alternaria (mold allergen).

Immunoblot and SPT (Table 5) showed homogeneous moderate levels of concordance were obtained for the dust allergens *Dermatophagoides pteronyssinus* and *Dermatophagoides farinae*, with kappa values of 0.598 and 0.576, respectively. These two allergens were the only ones that showed significant concordance. For the remaining allergens, although no statistically significant association could be identified, the concordances ranged from negative to null.

## 4. Discussion

Given the variety of available tests differing in speed of results and price, concordance data could be essential to assist physicians in selecting the appropriate options. This research provides results that aim to contribute to this knowledge through data obtained in a private medical center in the city of Arequipa, Peru.

Although there is insufficient scientific evidence to establish an official allergen sensitization profile in Peru, a study on allergen sensitization rates conducted in two cities, including Arequipa, identified *Cladosporium herbarum* and *Alternaria alternata* as mold allergens and *Olea europaea* as a pollen allergen present in Arequipa [16]. While the panel of allergens evaluated in that investigation was not extensive, these first two allergens mentioned above were utilized in the analysis for concordance in this study, as well as the three allergens for sensitization testing.

Focusing solely on the positivity levels in the aforementioned study conducted in Arequipa, which used the SPT, we can observe that our research found higher positivity rates for the three allergens evaluated. *C. herbarum* showed 47.22% positivity compared to 5.71% in the previous study, Alternaria showed 33.00% compared to 11.43%, and *O. europaea* or Olive showed 36% compared to 34.3%. These differences could be attributed to factors such as the inclusion of two different populations and a larger sample size of 200 individuals, of which 100 came from the Arequipa region. Additionally, variations in the age distribution of participants may have contributed to the different results. While the previous study included children as young as 4 years old, similar to our research, it also included individuals up to 70 years old [16].

The results obtained in this research reveal discrepancies in allergen sensitization rates between SPT, Immunoblot, and ALEX-2. However, within these discrepancies, ALEX-2 showed better concordance with SPT for the seven allergens identifiable by all three tests, with agreement found in five of the allergens. In contrast, ALEX-2 and Immunoblot, as well as SPT with Immunoblot, identified agreement in only two allergens out of the seven.

Among the tested allergens, particularly dog epithelium and cat epithelium, exhibited the highest positivity rates across all three diagnostic tests, with ALEX-2 demonstrating notably superior sensitization rates compared to the SPT and Immunoblot. For instance, in the case of dog epithelium, ALEX-2 yielded positive results in almost twice as many cases as the Immunoblot and substantially differed from the SPT. Of these cases, 62.86% were positive for ALEX-2, 34.29% for SPT, and only 5.71% for Immunoblot.

In some cases, SPT showed higher positivity rates, particularly with allergens such as dust mites from *D. ptenonyssinus*, pollen allergies from olive trees, and mold allergies like Cladosporium. Nevertheless, it is important to note the distinction between potential sensitivity to the allergen and the actual allergic reaction obtained through the SPT, as misinterpretation could lead to false positives [17, 18]. Additionally, the SPT is a procedure whose positivity can vary depending on the concentration of the allergen used [19]. When interpreting the results, it is crucial to consider the type of allergic respiratory disease. Higher levels of IgE, which can be measured through tests such as the Immunoblot, are more closely associated with asthma, whereas skin reactivity detected by the SPT is more commonly linked to AR [20].

As mentioned previously, three allergens could not be analyzed for concordance in the study, despite their high sensitivity, as they are not included in the Peruvian Immunoblot panel. These allergens have already been described in several studies in the pediatric setting and have already reported high levels of sensitization. For example, a study conducted in Jordan reported a sensitization rate of 18% for olive pollen [21], while Blomia tropicalis showed a sensitization rate of 47.7% in Peru [22], and grasses demonstrated a sensitization rate of 48% in Spain [23].

In patients with sensitization to allergens not included in the Immunoblot panel, such as olive pollen, ALEX-2 and SPT tests could be more useful to identify relevant allergens and guide treatment. In addition, identification of discrepancies between allergy tests could alert clinicians to the need for further evaluation to avoid misdiagnosis and provide optimal treatment.

The different levels of agreement found between the analyses showed differences between the three tests for several allergens, and because of these variations it also makes us consider that the concordance of the specific allergens is more important than the overall test concordance. While certain allergens showed substantial agreement across the tests, others displayed moderate or minimal agreement. For example, concerning Dermatophagoides mites, the agreement between ALEX-2 and the SPT presented a kappa of 0.822, suggesting nearly perfect agreement, whereas, for the common fungus Alternaria, the kappa agreement was 0.545, indicating moderate agreement.

When comparing ALEX-2 with Immunoblot, various levels of agreement were observed. The dust allergen *D. farinae* showed fair to substantial agreement, while mold allergens such as Alternaria demonstrated moderate agreement. Unfortunately, the authors are not aware of any other studies outside of the one presented in this paper that have directly compared these two diagnostic tests. However, in a study analyzing the concordance between ALEX-2 and ImmunoCAP ISAC, another reliable method [24], a total agreement was reached in 94% of cases, contrasting significantly with this study, where the agreement was found in only two out of the seven allergens evaluated, giving a percentage of less than 30%.

Regarding the concordance between SPT and Immunoblot, it is important to highlight that the dust mite allergens *D. pteronyssinus* and *D. farinae* were the only ones that showed a moderate concordance in both cases (kappa values of 0.598 and 0.576, respectively). This suggests good accuracy of Immunoblot in detecting sensitivity to dust mites. Our results are consistent with those of Wongpiyabovorn et al. [25], who observed a strong correlation between dust mites when evaluating Immunoblot with SPT; however, they found a correlation in 9 out of 10 types of allergens, unlike 2 out of 7 in our research. It should be noted, however, that this research also evaluated food allergies in this 10 allergen concordance analysis [25].

The main strength of this research lies in its relevance and novel contribution, as ALEX-2 is relatively new to the market, and limited research has been conducted on its positivity. Additionally, this study appears to be the first to focus on the concordance between these three diagnostic methods: SPT, Immunoblot, and ALEX-2.

However, this study also has limitations that should be acknowledged when interpreting the results. Because ALEX-2 is a new diagnostic test, there is limited published research available for comparative analysis. Additionally, the sample size and the exclusive performance of tests in a private medical center make it challenging to generalize results to a larger population. Although a systematic random sampling method was utilized, a larger sample would have strengthened the robustness of the results. Furthermore, while various data collection techniques, such as epidemiological questionnaires and allergy testing, were employed, the lack of long-term follow-up of patients might limit the complete understanding of the evolution of AR in this specific population.

In conclusion, this study presented an analysis of the concordance between SPT, Immunoblot, and ALEX-2 diagnostic methods for AR. The results underline the importance of generating more evidence to understand variations in sensitization rates and to enable the treating physician to make a better decision. ALEX-2 demonstrated higher sensitization rates for certain allergens compared to traditional methods such as SPT and Immunoblot, as in the case of dog and cat epithelium. In addition, a higher concordance between SPT and ALEX-2 was found.

## Figures and Tables

**Table 1:** Sociodemographic and clinical characteristics of patients with allergic rhinitis seen in a private center in Arequipa, 2023.

Demographic and clinical characteristics	*n*	%
Demographic variables		
Age (M-IQR)	9	(6–30)
Male	21	60.00
Female	14	40.00
Severity of allergic rhinitis		
Mild intermittent	10	28.57
Moderate–severe intermittent	5	14.29
Mild persistent	8	22.86
Moderate–severe persistent	12	34.29
Treatment adherence		
Compliance with treatment	13	37.14
Medical history		
Atopic dermatitis	5	14.29
Conjunctivitis	13	37.14
Asthma	5	14.29
Food allergy	0	0.00
Drug allergy	1	2.86
Family history		
Allergic rhinitis	30	85.71
Conjunctivitis	5	14.29
Atopic dermatitis	4	11.43
Asthma	3	8.57
Food allergy	1	2.86
Drug allergy	1	2.86
Treatment		
Intranasal corticosteroids	6	17.14
Antihistamines	18	51.43

**Table 2:** Results of prick test, Immunoblot, and ALEX-2 in the detection of respiratory allergens in patients with allergic rhinitis seen in a private center in Arequipa, 2023.

Allergen	Prick test positive (*n*, %)	Immunoblot positive (*n*, %)	ALEX-2 positive (*n*, %)
Food allergens			
Egg white	—	7 (20.00)	—
Apple	—	6 (17.14)	—
Wheat	—	1 (2.86)	—
Strawberries	—	1 (2.86)	—
Amaranth	—	—	6 (17.14)
Banana	—	—	1 (2.86)
Respiratory allergens			
Dust mites			
*D. ptenonyssinus*	6 (17.10)	6 (17.14)	13 (37.14)
*D. farinae*	8 (22.86)	7 (20.00)	6 (17.14)
Blomia	4 (11.43)	—	6 (17.14)
Periplaneta	4 (11.43)	—	—
Pollen			
Gramineas	4 (11.43)	—	10 (28.57)
Cypress	—	—	2 (5.71)
Roses	—	1 (2.86)	—
Olive	12 (34.3)	—	6 (17.14)
Oak	—	—	2 (5.71)
Salsola	—	—	7 (20.00)
Birch	—	—	4 (11.43)
Ragweed	—	—	4 (11.43)
Mugwort	—	—	5 (14.29)
Mold			
Alternaria	4 (11.43)	4 (11.43)	6 (17.14)
Cladosporium	2 (5.71)	1 (2.86)	1 (2.86)
Animals or insects			
Dog epithelium	12 (34.29)	2 (5.71)	22 (62.86)
Cat epithelium	9 (25.71)	4 (11.43)	18 (51.43)
Blatella	1 (2.86)	1 (2.86)	1 (2.86)
Pig	—	1 (2.86)	—
Rabbit	—	1 (2.86)	—
Horse	—	1 (2.86)	—
Bird feathers	—	1 (2.86)	—
Hamster	—	1 (2.86)	—

**Table 3:** Concordance of ALEX-2 (AL2) and skin prick test (SPT) in the detection of respiratory allergens, Arequipa-2023.

Allergen categories	AL2−SPT−	AL2−SPT+	AL2+SPT−	AL2+SPT+	Kappa	*p*
Dust allergens						
*D. ptenonyssinus*	21	1	8	5	0.381	0.010
*D. farinae*	27	2	0	6	0.822	<0.001
Mold allergens						
Alternaria	28	1	3	3	0.536	0.001
Cladosporium	32	2	1	0	−0.04	0.803
Animals and insect allergens						
Blatella	34	0	0	1	0.999	<0.001
Dog epithelium	11	2	12	10	0.260	0.070
Cat epithelium	17	0	9	9	0.493	0.001
Total	195	8	39	38	0.403	<0.001

**Table 4:** Concordance of ALEX-2 (AL2) and Immunoblot (IB) in the detection of respiratory allergens, Arequipa-2023.

Allergen categories	AL2-IB−	AL2-IB+	AL2+IB−	AL2+IB+	Kappa	*p*
Dust allergens						
*D. ptenonyssinus*	21	1	8	5	0.381	0.100
*D. farinae*	27	2	1	5	0.717	<0.001
Mold allergens						
Alternaria	28	0	3	3	0.545	<0.001
Cladosporium	33	1	1	0	−0.029	0.862
Animals and insect allergens						
Blatella	33	1	1	0	−0.029	0.862
Dog epithelium	13	0	20	2	0.069	0.263
Cat epithelium	16	1	15	3	0.105	0.316
Total	171	6	49	18	0.571	<0.001

**Table 5:** Concordance of prick test and Immunoblot, in the detection of respiratory allergens, Arequipa-2023.

Allergen categories	SPT−IB−	SPT−IB+	SPT+IB−	SPT+IB+	Kappa	*p*
Dust allergens						
*D*. *ptenonyssinus*	27	2	2	4	0.598	<0.001
*D. farinae*	25	2	3	5	0.576	0.001
Mold allergens						
Alternaria	28	3	3	1	0.167	0.268
Cladosporium	32	1	2	0	−0.400	0.803
Animals and insect allergens						
Blatella	33	1	1	0	−0.290	0.862
Dog epithelium	23	0	10	2	0.208	0.440
Cat epithelium	24	2	7	2	0.178	0.238
Total	192	11	28	14	0.689	<0.001

## Data Availability

The data used to support the findings of this study are available from the corresponding author upon request.

## Additional Points

This study evaluates the differences in respiratory allergen sensitization rates between three diagnostic tests that could improve diagnosis and allow more accurate therapeutic decisions to be made. The ALEX-2 multiplex allergy test showed better agreement with SPT compared to the Peruvian Immunoblot panel in identifying sensitization to respiratory allergens in patients with AR.
